# Label-Free Multiphoton Microscopy: Much More Than Fancy Images

**DOI:** 10.3390/ijms22052657

**Published:** 2021-03-06

**Authors:** Giulia Borile, Deborah Sandrin, Andrea Filippi, Kurt I. Anderson, Filippo Romanato

**Affiliations:** 1 Laboratory of Optics and Bioimaging, Institute of Pediatric Research Città della Speranza, 35127 Padua, Italy; filippo.romanato@unipd.it; 2 Department of Physics and Astronomy “G. Galilei”, University of Padua, 35131 Padua, Italy; deborah.sandrin@unipd.it (D.S.); andrea.filippi.5@studenti.unipd.it (A.F.); 3 L.I.F.E.L.A.B. Program, Consorzio per la Ricerca Sanitaria (CORIS), Veneto Region, 35128 Padua, Italy; 4 Crick Advanced Light Microscopy Facility (CALM), The Francis Crick Institute, London NW1 1AT, UK; kurt.anderson@crick.ac.uk

**Keywords:** multiphoton microscopy, label-free, second harmonic generation, third harmonic generation, quantitative imaging

## Abstract

Multiphoton microscopy has recently passed the milestone of its first 30 years of activity in biomedical research. The growing interest around this approach has led to a variety of applications from basic research to clinical practice. Moreover, this technique offers the advantage of label-free multiphoton imaging to analyze samples without staining processes and the need for a dedicated system. Here, we review the state of the art of label-free techniques; then, we focus on two-photon autofluorescence as well as second and third harmonic generation, describing physical and technical characteristics. We summarize some successful applications to a plethora of biomedical research fields and samples, underlying the versatility of this technique. A paragraph is dedicated to an overview of sample preparation, which is a crucial step in every microscopy experiment. Afterwards, we provide a detailed review analysis of the main quantitative methods to extract important information and parameters from acquired images using second harmonic generation. Lastly, we discuss advantages, limitations, and future perspectives in label-free multiphoton microscopy.

## 1. Introduction

Light microscopy is a gold standard technique in biomedical research and clinical diagnosis [1]. The huge technical developments toward more and more sophisticated apparatus [2,3,4] enhanced the broad use of light microscopy, which in turn increased the need for innovative microscopy approaches. Some examples of these multidisciplinary solutions are nonlinear optical microscopy, super-resolution [5,6,7], fluorescent markers, and optimization of sample preparation [8,9]. Confocal laser scanning microscopy is routinely used in biomedical research because it ensures high resolution and high contrast compared to epifluorescence microscopes (for a complete review, see Jonkman et al. [10]). A drawback of this technique is the limited penetration into the sample (50–100 µm) and the photodamage caused by illumination light, which is usually in the range of 400–600 nm. Thanks to the use of longer wavelengths (800–1200 nm), multiphoton microscopy reduces sample damage and provides deeper penetration (250–500 µm) into the specimen, although with some loss of resolution. This technique has found success as a non-invasive imaging tool for thick biological tissues and living animals. There are typically two labeling strategies for multiphoton experiments. Fluorescent proteins may be expressed under genetic control throughout a tissue sample; however, this requires complex and time-consuming genetic manipulation of the model organism genome. Alternatively, conventional antibody labeling strategies may be employed; however, the penetration of probes into tissue is not straightforward and may require heavy detergent action, which can disrupt tissue ultra-structure. Of notice, any external operation may alter the intrinsic characteristic of the specimen. For this reason, the possibility to analyse biological samples without labeling procedures while maintaining molecular specificity is becoming more and more popular.

Label-free microscopy methods rely on photophysical processes to generate signals through specific interactions with biological molecules and offer great potential for basic research and clinical applications. Multiphoton microscopy is probably the most popular label-free technique. Using the same optical path, it can identify three different types of signal: autofluorescence, second harmonic generation and third harmonic generation. Two-photon autofluorescence has been widely used to identify and quantify metabolic molecules such as NADH and tryptophan [11,12,13]. Using fluorescence lifetime imaging (FLIM), it is even possible to quantify the proportion of NADH that is free or protein-bound [14] and distinguish NADH from NADPH [15,16,17]. In contrast, second and third harmonic signals (SHG and THG) are non-fluorescent photo-physical conversions dependent on the intrinsic properties of the target biomaterial [7,18]. A variety of molecules have been reported to generate second harmonic signals, such as collagen, myosin, microtubules, silk, starch and cellulose. All these molecules are characterized by non-centrosymmetric architecture or hyperpolarizability, making them SHG active molecules (also named harmonophores) [19,20]. In SHG microscopy, two photons of the same frequency pass through these biomaterials and result in a photon with doubled frequency (and thus half wavelength). Otherwise, third harmonic generation requires the presence of an interface characterized by remarkably different refraction indexes that cause a symmetry break or by molecules with third order nonlinear susceptibility. Some examples of THG sources are lipid droplets, elastin fibers, bone calcifications, and cellular membranes [21].

**Figure 1:**
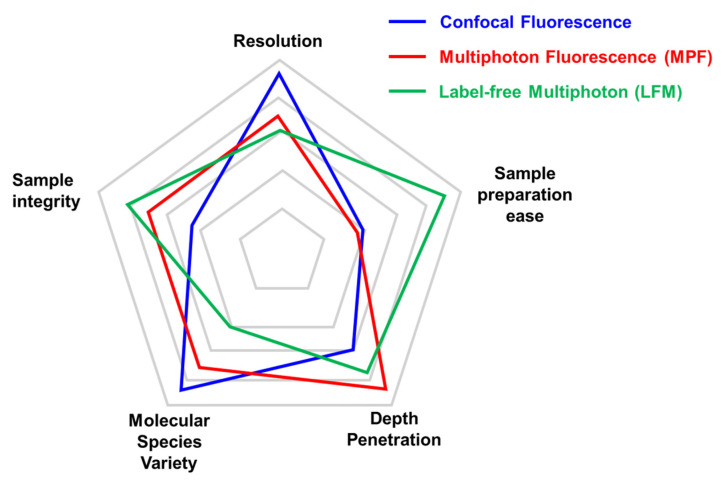
Comparison of key parameters of different imaging techniques. Each microscopy technique has strengths and weaknesses that are important to consider before choosing the adequate microscopy approach. We limited the radar plot to five parameters (Resolution, Sample preparation ease, Depth penetration, Molecular species variety, and Sample integrity), although experimental needs may require considering other aspects (i.e., acquisition speed). The relative performance of confocal microscopy (in blue), multiphoton (in red), and label-free multiphoton (in green) are compared with the outer position indicating the best performance for that parameter. The same set-up can be optimized for specific approaches (multiphoton fluorescence vs. label-free multiphoton).

A comparison between different microscopy approaches can vary depending on the specific application (Figure 1). For the analysis of structural components within tissues, label-free multiphoton (LFM) microscopy can be successfully used to retrieve information in a staining-free fashion. This results in a great advantage in terms of sample preparation ease where the absence of immunofluorescence or fluorescent protein expression simplifies benchwork protocols. On the other hand, despite the high signal specificity obtained by SHG and THG, the molecular species that can be monitored in LFM are limited. In multiphoton fluorescence (MPF) and, even more, in confocal fluorescence microscopy, a huge number of antibodies and fluorescent tags have been developed to mark the protein of interest, overcoming this limitation. Confocal microscopy, based on visible wavelengths, possesses higher resolution compared to MPF and LFM that use longer excitation wavelengths. Moreover, LFM is more sensitive than MPF to a degradation of the focal spot caused by scattering inside the sample that reduces conversion efficiency [22]. For the same reason, LFM depth penetration is slightly reduced compared to MPF on the same sample. Lastly, sample integrity can be estimated in terms of photobleaching and phototoxicity during imaging. In LFM, photobleaching does not occur for SHG and THG molecules, while it has to be considered for fluorescent dyes and proteins.

Other label-free imaging techniques have been developed and are based on a variety of optical approaches. Holotomographic microscopy and spiral phase microscopy are sensitive to variations of the refractive index of cellular compartments and have been used to study organelle dynamics [23] and enhance edge contrast [24]. Raman microscopy can identify chemical bonds and is particularly interesting to quantify lipids abundance. Variants of Raman microscopy include CARS (Coherent Anti-Stokes Raman Spectroscopy) and SRS (Stimulated Raman Spectroscopy). Both achieve a better signal to noise ratio than Raman microscopy and are amenable to imaging small molecules that are not easy to tag with a fluorophore. Photoacoustic imaging exploits the conversion of incident laser light into heat by the tissue to reconstruct an image [25,26]. The optical resolution of this technique is low compared to optical microscopy; however, it achieves deeper penetration (several millimeters). Lastly, interferometric scattering (iSCAT) microscopy is a light scattering technique that is sensitive and can be used to determine the molecular weight of single unlabeled proteins released by living cells [27,28].

Here, we will focus on label-free multiphoton microscopy with interests in biological and clinical applications as well as data analysis systems developed in recent years. Moreover, we will discuss future implementations of multiphoton microscopy that would help to increase resolution and depth penetration in terms of component technical developments and sample optimization.

## 2. Multiphoton Technique

Multiphoton microscopy (MPM) encompasses several laser-scanning methods based on the nonlinear interaction of light with the specimen. In this context, “nonlinear” means that the intensity of the signal depends on the simultaneous interaction of the probe with two or three photons [29]. In single photon microscopy, photons at a determined wavelength can excite the target fluorophore, delivering the adequate amount of energy to induce the transition to the excited state (S1). Then, the fluorophore relaxes back to the ground state (S0) emitting a photon of reduced energy and longer wavelength. In MPM, the use of longer wavelengths comes with photons of halved energy (for two-photon microscopy). For this reason, to obtain the excitation of the fluorophore to the S1 state, two exciting photons are needed. The simultaneous interaction with more than one photon requires an extremely high density of photons at the focal point of the objective, which is typically achieved using pulsed lasers producing mega-watts to giga-watts of power. The optical sectioning capability of MPM results from the selective generation of a signal in the focal plane of the objective. This approach enables the collection of all emitted photons, even the scattered ones, without the need for pinhole filtering used in confocal microscopy, since all signals are generated in the focal volume [1]. Moreover, the longer wavelengths used in MPM face less scattering and absorption by biological matter, allowing reaching deeper layers inside the sample. Therefore, MPM represents the best non-invasive technique to achieve imaging in deep explanted tissues or living animals [1,30]. The advantage of the volume-confined excitation is that the lack of out-of-focus excitation in two-photon excitation (TPE) reduces specimen photobleaching in non-imaged areas. This also means that photodamage is highly confined, allowing long-term observations of biological specimens that would otherwise be limited using single photon excitation. Of notice, in the focal volume, the illumination power must be controlled carefully. Photobleaching and photodamage are also nonlinear phenomena where a higher order of photon absorption has been observed [31].

Thanks to its high-resolution capabilities, MPM has provided unprecedented possibilities for the study of neuroscience [32], metastasis [33], calcium imaging [6], embryonic development, and cell–cell in vivo interactions.

**Figure 2:**
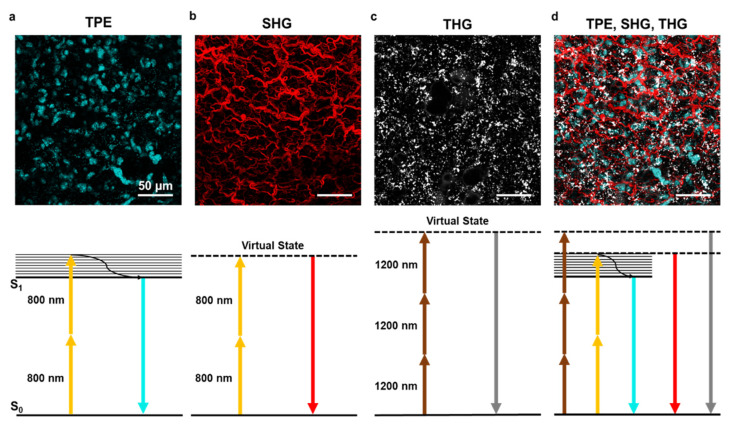
Two-photon excitation, second and third harmonic generation. (**a**) Example of two-photon excitation (TPE) of DAPI stained nuclei in lung tissue. Bottom panel shows the corresponding Jablonski diagram using 800 nm excitation wavelength. (**b**) Second harmonic generation (SHG) signal elicited with 800 nm wavelength on lung tissue collagen structure. The bottom panel shows the corresponding Jablonski diagram using 800 nm excitation wavelength. (**c**) Third harmonic generation (THG) signal of lipid bodies in lung tissue using 1200 nm wavelength. The bottom panel shows the corresponding Jablonski diagram. (**d**) A combination of TPE, SHG, and THG can be obtained using two laser sources at different wavelengths. Images obtained with the 800 nm source are merged with THG obtained with the 1200 nm source. The scale bar is 50 µm for all images.

The most common MPM variation is two-photon excitation (TPE) microscopy [30] (Figure 2a). This technique is based on the simultaneous adsorption of two photons by a fluorophore in a single quantum event. This phenomenon was theoretically predicted by Maria Goeppert-Mayer in 1931 but could be experimentally verified only after the advent of lasers. In 1961, Peter Franken and colleagues demonstrated the frequency doubling of light focusing a ruby laser on a quartz crystal [34]. The development of ultrashort pulsed Ti:Sapphire mode-locked lasers allowed more practical generation of the necessary photon density. Exploitation of two-photon laser excitation laid dormant until Denk et al. devised a practicable two-photon laser-scanning fluorescence microscope [30] that has opened new frontiers in biomedical research.

The occurrence of the double-absorption event is determined by photon–molecule interactions, which must occur with a relative delay shorter than the typical virtual state lifetime of a given fluorophore [30]. Furthermore, the two-photon excitation rate depends on the second power of the incident light intensity and is ≈10–14 times smaller than the one-photon absorption rate; hence, the successful implementation of TPE imaging requires extremely high photon fluxes, which in practice translates into the use of mode-locked laser sources with pulse durations below 1 *ps* and frequencies of about 100 MHz. Ti:Sapphire lasers ensure adequate tunable sources in the 760–960 nm window and are the most used lasers for MPM. In the last years, the commercial availability of laser sources has grown, opening to new wavelength windows beyond the Ti:Sapphire range, with the advent of optical parametric oscillators (OPO) whose typical spectral range is 1030–1300 nm. An additional option is offered by ultrashort pulsed lasers, which can operate at specific wavelengths, such as 775, 1030–1060, and 1500 nm. The combination of these two laser sources allows multimodal excitation of the sample together with wavelength mixing that consists of the simultaneous absorption of two different wavelengths enabling different state transitions. This resulted in an optimal configuration for the excitation of certain fluorophores, such as YFP, whose absorption spectra is centered outside the Ti:Sapphire emission laser [35]. The acquisition wavelengths depend on the setup design but usually range from 380 to 700 nm, ensuring proper separation of excitation and emission signal.

Despite the need for very intense light sources, TPE presents several advantages over classical single-photon techniques: the wavelengths used are in the near-IR, making them less subjected to scattering or absorption from biological thick specimens (which means deeper penetration capabilities) [36]; due to the high photon flux required to achieve the two-photon absorption, the focal volume is very small, about 0.1 μm^3^ [37]. Such a small focal volume is the main reason for the inherent ability of MPM to perform axial sectioning of samples and for the photobleaching restricted to the focus plane [38]. The effective spatial confinement is a direct consequence of the insignificant single-photon fluorescence excited in out-of-focus regions. Note that TPE can be used to generate fluorescence from all possible fluorophores, including fluorescent proteins, chemical dyes, and autofluorescence.

The use of different excitation wavelengths combining the Ti:Sapphire laser with an OPO allows multiplexed fluorescence and label-free analysis of the same sample, as shown in Figure 2d on a lung ex vivo sample.

## 3. Label-Free Multiphoton Microscopy in Biomedical Research

A special form of MPM that is becoming more successful in the biomedical context is label-free microscopy. The combined imaging of autofluorescence with second and third harmonic generation (SHG and THG) provides molecular information about the sample without any staining procedure.

### 3.1. Autofluorescence

The autofluorescence signal is generated by molecular components of cells or matrices and can be used to reconstruct tissue morphology without any staining. Endogenous sources of autofluorescence signals are metabolic substrates (e.g., NADH and FAD), structural proteins (e.g., elastin and keratin), lipofuscins, and melatonin. This means that active cells are perfect sources of autofluorescence signals. A peculiar application of autofluorescence imaging can be found in regenerative medicine. In this biomedical field, tissues from animals or human biopsies are decellularized to constitute ex novo scaffolds for induced pluripotent stem cells (iPS) repopulation or patient-derived 3D models [39,40]. The success of the decellularization procedure is confirmed by DNA quantification as a gold standard reference; however, the presence or absence of cellular components can be assessed by autofluorescence imaging with the advantage that the analyzed tissue remains intact and can be used for other purposes. Moreover, autofluorescence from elastin or other structural proteins gives useful information on scaffold integrity and preservation. On the other hand, autofluorescence signals from metabolic substrates, such as NADH, can be efficiently used to discriminate cellular activity. For example, in muscle biopsies, NADH autofluorescence intensity correlates with the metabolic state of the fibers [7].

### 3.2. Second Harmonic Generation

Second harmonic generation is a nonlinear coherent light-scattering phenomenon, resulting from the interaction of light waves with molecular structures that have specific crystal-like physical properties. SHG results from the conversion of two incoming photons into one emitted photon having twice the energy and therefore half the wavelength. An SHG signal can be obtained with both Ti:Sapphire and OPO lasers at any wavelength from 800 to 1200 nm. The dependence of SHG signal on incident light intensity must be considered in LFM imaging when using Ti:Sapphire lasers and OPO that display an intensity profile peaked at shorter wavelengths (around 800 nm) and then decline toward longer wavelengths, and it has been demonstrated by Campagnola and colleagues that shorter wavelengths are more efficient in SHG microscopy [22]. The emitted signal is generally collected in transmission due to the momentum and energy conservation properties of the generated SHG signal. Moreover, molecules such as collagen and starch allow collection of the SHG signal both in reflection or transmission. For collagen, this feature has been used to obtain a quantitative evaluation of fibrillar structure [41]. The SHG signal collected in transmission is known as a forward signal, which is more intense than an epicollected signal, which is named backward SHG. SHG allows the identification of molecules with non-centrosymmetric structure via interaction with incident light. In this way, the intrinsic properties of the biological tissue can be studied with no need for contrast-enhancing or labeling while providing high-resolution 3D label-free reconstruction of the imaged portion. The second harmonic generation has been applied to a plethora of samples ranging from zebrafish embryos to bone, fat and skin tissue, brain, heart, and muscles. In all these tissues and organs, some features are particularly efficient at generating second harmonic signals. Of note, collagen is very effective in SHG, resulting in one of the most frequently analyzed matrix components in label-free microscopy (Figure 2b). Another SH generator is myosin, which is a key protein in cardiac and muscular tissues. Some protocols to discriminate between the two have been developed and are mainly based on polarization setup.

### 3.3. Third Harmonic Generation

The third harmonic signal is generated at interfaces and structural inhomogeneities through the interaction with incident light. THG occurs at structural interfaces [21] such as boundaries of regions with highly different refractive indexes. Lipid droplets are particularly efficient THG structures [42] (Figure 2c); moreover, elastin fibers, bone calcification, and cellular membrane can be visualized with this technique. While every biological specimen is extremely rich in interfaces, thus being a perfect theoretical candidate for THG, in practice, THG imaging is much less applied and requires a highly specialized multiphoton microscope with long laser wavelengths. THG signals occur at one-third of the illumination wavelength; for example illumination at 900 nm would produce a signal at 300 nm. However, detection in the UV range is compromised by the high absorbance of this wavelength in any biological tissue. Practically, the range 380–450 nm is well suited for THG imaging but requires the availability of longer wavelength lasers (or OPO) in the region above 1050 nm. This ensures minimal absorption and overlap with signals in green and red fluorophores spectral regions, as well as SHG detection at 530–600 nm. Last but not least, THG efficiency is sensitive to laser power, and even a small loss of photons in the focal region may compromise imaging quality. In deep tissues, THG signals are affected by light scattering and aberrations introduced along the optical path within the sample.

### 3.4. Research and Clinical Applications

The success of label-free multiphoton imaging is well represented by its wide use in many diverse fields from regenerative medicine to cancer and embryogenesis. Table 1 reports a representative overview of application fields and results obtained thanks to label-free multiphoton microscopy both at the basic research level and clinical diagnosis. Label-free microscopy started as a technically demanding method for experienced users. However, the reduced cost and improved stability of pulsed lasers led to the increased availability of user-friendly systems and the application of nonlinear imaging methods in many biomedical fields. More recently, proof-of-concepts development of the technique is moving from imaging bench to clinical application in optical biopsies. An excellent example of clinical application is DermaInspect, which is a high-resolution multiphoton microendoscopy used in clinical practice to identify melanoma lesions [43,44].

## 4. Sample Preparation

Sample preparation is a crucial point in microscopy where every single step may introduce artefacts and distortions. For tissue samples, key steps in fluorescence microscopy preparation are fixation, sectioning, permeabilization, labeling, and mounting. All these steps are discussed in detail for confocal microscopy by Jonkman et al. [10]. Sample preparation for label-free microscopy can be simple due to the absence of permeabilization and labeling steps, unless of course a combination of label-free and immunofluorescence staining is required. In the following subsections, we will briefly discuss each step in the label-free context.

### 4.1. Fixation

Every tissue sample requires optimization of the fixation protocol that depends on tissue characteristics and experimental needs. Fixation can be physical or chemical. Physical fixation is more used for electron microscopy; however, samples snap-frozen in liquid nitrogen is an option. While for some samples, reduced preservation has been observed, other tissues, such as muscles from rodents, can be processed with the snap-frozen technique and still maintain their structure as well as autofluorescence signal from NADH. On the other side, chemical fixation is widely used and uses a cross-linking approach, typically with formaldehyde (FA) and the corresponding para-formaldehyde (PFA). However, the timing, percentage, and temperature of fixation protocols require a sample-specific optimization. Another chemical fixation protocol employs precipitation methods (methanol or acetone) and is a good alternative for all those antibodies that do not work in the presence of aldehyde fixatives. Indeed, the fixation procedure must accommodate the adequate balance between efficient cross-linking and maintenance of antigenicity in case staining is required. Moreover, an excess of fixative may result in unwanted autofluorescence signal [68] or overshadowing of expressed fluorescent proteins such as green fluorescent protein, GFP. A detailed analysis of the effect of fixation on SHG has been proposed [69], demonstrating that while tubules are highly compromised, collagen and myosin are not sensitive to PFA fixation. A universal protocol for fixation does not exist; however, one of the advantages of label-free multiphoton microscopy is that samples can be freshly excised and immediately imaged under the microscope without fixation procedures, thereby eliminating the possibility of fixation-induced structural artefacts. However, the viability of unfixed tissue is limited, and it may deteriorate during the experiment.

### 4.2. Sectioning

The sectioning step is not mandatory in label-free multiphoton microscopy; however, in the context of clinical samples, sections are widely used. The sectioning procedure can be divided into cryosectioning and paraffin-embedded microsections. In both cases, advantages and disadvantages can be found. Sections from paraffin-embedded samples can be preserved for longer times and maintained at room temperature, while cryosections require −80 °C maintenance. Usually, sections from paraffin-embedded [70] samples are thinner (3–10 μm) compared to cryosections (10–30 μm), and this results in a reduced backscattered SHG signal. However, a strong improvement can be obtained by placing a reflective mirror under the glass slide holding the sample. To further enhance the signal from paraffin-embedded sections, the rehydration procedure has been shown to increase both the contrast and intensity of the SHG signal. Last but not least, working with sections instead of thick samples makes staining with antibodies that show a difficult diffusion inside the tissue after a few tens of micrometers more amenable.

### 4.3. Mounting

The last step before imaging consists of mounting the sample under the microscope objective. Modern high-resolution objectives are generally designed to work with specific coverglass slides (# 1.5), in water dipping mode, or both. In the latter case, the objective has a ring collar that needs to be adjusted to the imaging conditions. Everything placed between the objective and the focal plane of the sample is crucial for imaging quality. Here, aberrations may be introduced by inappropriate coverslips or refractive index mismatch. Inverted microscopes position the objective below the sample, whereas upright microscopes position the objective above the sample. The first condition imposes the use of a coverslip between the objective and the sample. Upright microscopes may also be used with a coverslip, or the sample may be imaged in an open chamber using an objective dipped directly into the sample buffer. In the latter condition, the absence of refractive index variations and coverslips minimizes the spherical aberrations and improves laser focus toward the diffraction-limited minimum (≈325 nm in diameter). This condition is particularly interesting for label-free microscopy, since the absence of fluorescent dyes does not impose the use of a mounting medium to preserve the fluorophore from fading. Moreover, the same water-dipping approach can be applied to sections adherent on a glass slide without the need for further processing or deparaffination procedures.

## 5. Quantitative SHG Image Methods

**Figure 3:**
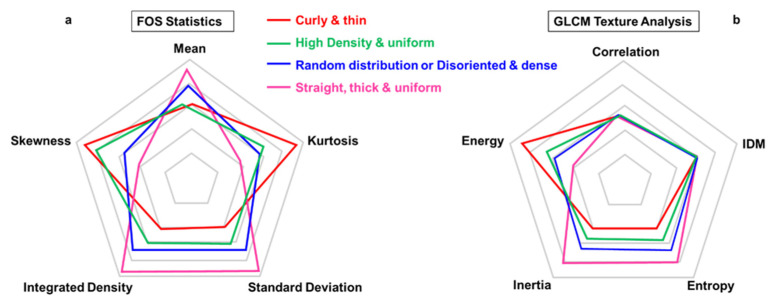
Radar plots for first-order statistics (FOS) and second-order statistics (gray level co-occurrence matrix, GLCM). Comparison of the statistical parameters between four categories of typical fibers arrangements: curly and thin (red), high density and uniform (green), random distribution or disoriented and dense (blue), and straight, thick, and uniform (magenta). (**a**) FOS provides the calculation of five statistical parameters: mean, kurtosis, skewness, integrated density, and standard deviation. Straight and thick fibers (magenta) are associated typically to higher values of mean, integrated density, and standard deviation. Differently, curly and thin fibers (red) are represented with higher kurtosis and skewness parameters, any intermediate situation can be shown by median values of the five FOS parameters (green and blue) [71]. (**b**) The second-order statistics (GLCM texture) is based on inter-pixel analysis, and the specific parameters are inverse difference moment (IDM), energy, inertia, entropy, and correlation. Correlation and IDM parameters do not show any significant trend according to the specific features of the tissue analyzed. On the other hand, energy shows higher values for curly and thin fibers (red), inertia and entropy are predominant for straight, thick, and uniform texture (magenta), intermediate values are represented by high density and random distribution (green and blue) [71].

**Figure 4:**
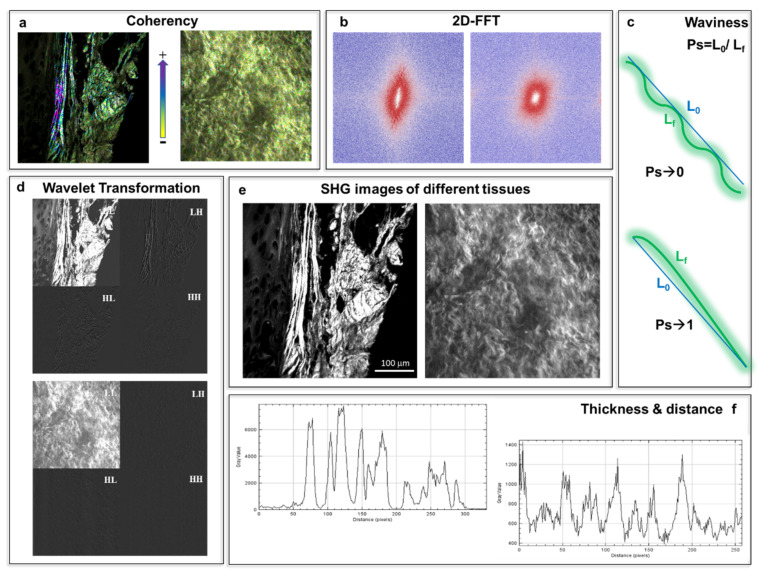
Analysis of SHG images of native and decellularized tissue (**e**) using ImageJ software. (**a**) Coherency analysis showing different values between 0 and 1 with 1 indicating a highly orientated structure. (**b**) Two-dimensional Fast Fourier Transformation (2D-FFT) images indicating a textural feature of the tissue show fibers with the same alignment. (**c**) The straightness parameter Ps quantifies the waviness of fibers and is bounded between 0 and 1. A bundle with Ps = 1 indicates a straight fiber; in contrast, Ps converges to zero when the fibers get very wavy. (**d**) Wavelet transform analysis applies a decomposition of the images (**e**), into four sub-images: low–low (LL), low–high (LH), high–low (HL), and high–high (HH) frequencies. LH, HL, and HH are three high-frequency sub-images that are horizontal, vertical, and oblique, respectively. LL is the low frequency sub-image that contains the main information of the decomposed image. (**f**) Plots profile generation, distances between the peaks can be associated with the distance between the fibers, and similarly, the widths of the peaks may give the thickness of them.

Most published label-free multiphoton imaging studies took advantage of SHG to describe biological tissue organization from a qualitative point of view. Quantitative approaches were implemented only in the last few years. In the section below, we give a simplified summary of the analysis methods and the corresponding interpretation that can be associated with the multiphoton images (see Figure 3 and Figure 4). The methods are classified according to the features of the fibers that are possible to study: amount, texture, orientation, waviness, thickness, and distance. Some image properties (i.e., texture description and fibers orientation) can be treated with different approaches to extract similar and complementary information. The microscopic analysis can be run with ImageJ (with the Fiji image processing package, https://imagej.net/Fiji) using different commercial or user-made plug-ins automatized with MATLAB and LabVIEW [18,52,71,72]. Table 2 summarizes the features and the approaches employed for image analysis and the corresponding degree of difficulty for each method according to the sophistication of the two-photon microscope and data analysis complexity.

Table 2 assigns a higher degree of complexity to the set-up employed for the forward–backward SHG signal approach and the polarization method. Indeed, in both cases, a more sophisticated two-photon microscope is needed by introducing components and different detection pathways (see Section 5.2). The wavelet transformation method is more complex, since it is still a novelty for texture analysis and not very well implemented in commonly available software.

### 5.1. Amount and Texture Description

#### 5.1.1. Intensity-Based Analysis

→ First-Order Statistics (FOS)

In the first-order statistics, the intensities of individual pixels are considered independently from their neighboring pixels. Every pixel receives a value that is proportional to the detected signal, which can be associated with the amount of the fibers. Five first-order parameters (mean, standard deviation, integrated density, skewness, and kurtosis) are useful for SHG imaging analysis [18,52,71] (see Figure 3a); the corresponding mathematical expression is described by Mustaço-Guidolin et al. and Haralick et al. [52,73]. The meaning and interpretation of the parameters are reported in Table 3.

According to Mostaço-Guidolin et al. [71], higher values of mean, integrated density, and standard deviation are associated with straight and thick fibers. Differently, curly and thin fibers are represented with higher kurtosis and skewness parameters. Any intermediate situation can be shown by median values of the five FOS parameters (see Figure 3a). It is crucial to mention that FOS analysis is very sensitive to laser power variations, and comparison between different samples can be difficult. Monitoring laser power during the experiments is mandatory.

→ Second-Order Statistics (Gray Level Co-Occurrence Matrix, GLCM)

The second-order statistics are based on inter-pixel correlation depending on the spatial arrangements of pixel intensities inside the region of interest. It is a measure of the probability of a pair of pixel values occurring at selected distances apart in the image, providing textural information for that region. This probability function is known as a co-occurrence matrix. Indeed, the most robust and frequently cited method for texture analysis is based on extracting various textural features from a gray level co-occurrence matrix (GLCM) [52]. The specific parameters most used are inverse difference moment (IDM), energy, inertia, entropy, and correlation, as reported in Table 4. In Image-J, a specific plug-in called “GLCM texture” permits such analysis, and the corresponding mathematical expressions are described by Mustaço-Guidolin et al. and Haralick et al. [52,73].

In Figure 3b, the radar plot shows the dependency of the GLCM parameters from the characteristics of the fibers. Correlation and IDM parameters do not show any significant trend according to the specific features of the tissue analyzed. Indeed, it is difficult to link some variations in the GLCM parameters directly to certain visual differences between the images [71].

In summary, first-order statistics (FOS) are parameters extracted directly from the original image while GLCM statistics are derived from a matrix that is built upon the inter-pixel correlation of the original image.

Overall, Mostaço-Guidolin et al. demonstrated that the use of the full set of calculated texture parameters (combined FOS and GLCM) gives the best classification accuracy, at least in skin disorders [71].

#### 5.1.2. Transform-Based Methods

→ 2D Fast Fourier Transformation (2D-FFT)

The transform-based texture analysis techniques convert the image into a new form using the spatial frequency properties of the pixel intensity variations allowing extracting textural characteristics from the image. Indhal and Næs [74] illustrated the use of two-dimensional Fast Fourier Transformation (2D-FFT) images for textural feature studies that can distinguish collagen fiber bundles with the same alignment. FFT has been by far the most used method to characterize SHG images due to its simplicity and availability in several image analysis software packages (see Figure 4b). FFT analysis can be useful when combined with forward–backward SHG signal or polarization-resolved SHG images, as it provides a quantitative measure of fiber orientation (see Table 2) [75,76,77].

→ Wavelet Transformation

Wavelet transforms have been preferred recently in image texture analysis due to their space-frequency decomposition abilities. This method has been used to characterize and treat the problems of texture segmentation and classification [78,79,80,81]. Wavelet texture analysis methods currently appear to be the most powerful approach to image texture examination [82]. Indeed, two-dimensional wavelet transforms perform a space-frequency decomposition, which is more suitable than the frequency decomposition provided by the 2D FFT. The wavelet transform decomposes an image into four sub-images: low–low (LL), low–high (LH), high–low (HL), and high–high (HH). LL is the low-frequency sub-image that contains the main information of the decomposed image. LH, HL, and HH are three high-frequency sub-images that are horizontal, vertical, and oblique, respectively (see Figure 4d). For an image with a coarse texture, the energy (from wavelet transformation) is mainly concentrated in the low-frequency sub-image, while for an image with a thin or complex texture, the energy is mainly concentrated in the high-frequency sub-image [82]. Nevertheless, it is complex to use due to the lack of a suitable and user-friendly implementation in common software. Further exploration of the capability of wavelets to help in the interpretation of SHG images is still needed.

### 5.2. Fibers Orientation

→ Forward–Backward SHG-Signal (F-SHG/B-SHG)

F-SHG/B-SHG ratio measurements can be useful for assessing the fiber orientation content [77,83]. Laterally oriented fibers appear primarily in the backward direction, whereas axially oriented fibers appear primarily in the forward direction [18]. To successfully extract information from this measure, the relative collection efficiencies of the two detection pathways (forward and backward), including the detectors, need to be calibrated for each objective/condenser combination [18,41]. Typically, previous studies comparing both signals have relied on quantifying the ratio of their intensities (F-SHG/B-SHG ratio) [37,84]. For example, the F-SHG/B-SHG ratio has been used to assess the thickness of fibril shells and the ionic strength of the surrounding medium [84]. In other studies, the dependence of the F-SHG/B-SHG ratio on both the scattering properties of the tissue and the focal depth within the sample has been reported [37]. Probably, F-SHG/B-SHG ratio measurements are one of the most common quantitative measures presented in SHG image analysis. However, more rigorous experiments with standardized samples must be performed.

→ Polarization

Polarization-resolved SHG is an alternative that can be used to extract information about the orientation of fibers in a certain image region; some authors presented examples of this type of SHG measurement [85,86,87,88]. The experiment can be in the form of measuring the intensity as a function of laser polarization or analyzing the signal anisotropy for constant linear polarization excitation [18,41].

In the first approach, at the beginning, the laser polarization is aligned with the long axis of collagen fiber(s), and then, images are typically acquired at least every 10° of laser polarization, through 180° of rotation. The intensities of the images are recorded at the starting point and after the rotation. This can be implemented by two methods, which are, in principle, physically equivalent. In the first, the specimen is fixed, and the polarization is rotated in the beam path with a λ/2 plate, or the second and more precise method is to place a polarization beamsplitting cube in the infinity space to select one linear polarization and then rotate the specimen for this excitation [41].

The second measurement determines the SH anisotropy by determination of SHG intensity detected after a laser polarizer oriented parallel and perpendicular to the laser polarization, respectively. Here, the linear polarization of the laser is fixed at 45° relative to the predominant fiber axis, and then in successive images, the SHG parallel and perpendicular components are measured relative to this excitation polarization [41].

At the molecular level, the polarization-sensitive second harmonic generation (PSHG) microscopy technique has been exploited by Psilodimitrakopoulos and colleagues [19] to uncover biological information non-accessible by intensity SHG analysis. In particular, using a pixel-level resolution analysis, they could retrieve polarization data on two different SHG active molecules (collagen and myosin) from the same image [20] or the helical pitch angle of amylopectin in starch [89].

→ Coherency (C)

The coherency parameter (C) permits estimating the local orientation of the fibers. The mathematical description for calculating C using OrientationJ, which is an ImageJ plug-in [72], is described by Rezakhaniha et al. [90]. Coherency is bounded between 0 and 1, with 1 indicating highly oriented structures and 0 indicating isotropic areas (see Figure 4a). Recently, our group and collaborators calculated the coherency parameters for collagen and elastin to verify the local dominant orientation in representative regions of interest for bovine and porcine pericardia before and after the decellularization process [39].

### 5.3. Fibers Waviness

To quantify the waviness of fibers, a straightness parameter Ps is used, which is defined as the ratio of the distance between two points of the collagen bundle and the corresponding length [90]. Ps is bounded between 0 and 1; a bundle with Ps = 1 indicates a straight fiber; in contrast, Ps converges to zero when the fibers get very wavy (Figure 4c). The quantification of the fiber waviness can be done by using the ImageJ plugin NeuronJ, as reported in our previous studies [39].

### 5.4. Fiber Thickness and Distance

Such analysis can be done by selecting a region in the image or by simply drawing a line. Subsequently, by creating a plot profile, it is possible to extract distances between the peaks that can be associated with the distance between the fibers, and similarly, the widths of the peaks may give the thickness of them (Figure 4f).

## 6. Limitations and New Perspectives

Label-free multiphoton microscopy publications have had an exponential growth in the last two decades, being applied to almost all biomedical research fields. Interestingly, a recent development of the technique is moving label-free microscopy toward clinical applications, opening new perspectives and expanding the field of histological analysis from ex vivo biopsies to in loco imaging.

There is no doubt that label-free multiphoton microscopy is a potent tool to study biological tissues due to the low invasiveness and phototoxicity of this technique. Indeed, in the last decade, the number of studies and publications exploded in different areas and applications (as reported in Table 1). Nevertheless, this technique presents some limitations.

First, the laser source and the microscope components are quite costly, therefore requiring a substantial initial investment. Second, even though it is greater than confocal microscopy, limited penetration depth (250–500 µm) makes the technique not useful for certain applications. During the last years, many efforts have been made to overcome such limitations. Optical clearing [91,92,93,94] methods are applied to biological samples to achieve transparent tissue allowing unprecedented three-dimensional views of enormous volumes of specimens. These methods employ dehydrating samples, extracting lipids and refractive index homogenization to a high value (presumably matching the refractive index of remaining proteins) by using, for instance, hydrogel embedding and/or organic solvent. Some residual problems need to be solved (especially sample swelling and/or shrinkage), but optical clearing is a good starting point to increase the penetration depth.

Another issue is the spatial resolution of multiphoton microscopy. Resolution is determined by the illumination wavelength, and using a longer wavelength results in an approximately 1.4× reduction in resolution compared to confocal microscopy. Subtractive SHG microscopy has been developed to enhance and contrast using a circularly polarized vortex beam. As it happens, for stimulated-emission depletion (STED) fluorophore-based microscopy [5], the excitation Gaussian beam is overlapped with a donut-shaped beam generated by a spatial light modulator to scan the same field of view [95]. The combination of the two images allows increasing the resolution of collagen fibers by a factor 1.3 [95]. Other methods for an increased spatial resolution include image-scanning microscopy [96,97]. In this approach, every single pixel of detection is considered a small pinhole, and signal collection can be performed via rescanning microscopy or using an emission-side galvanometric mirror (galvo) to double the distance between adjacent scan points before image acquisition. Another option to improve optical resolution, by a factor of 1.7, is the installation of AiryScan detectors instead of standard gallium arsenide phosphide (GaSP) [3]. Moreover, the improvement of the microscope performance can be done by acting directly on the sample, where exceptional results have been obtained with expansion microscopy [9]. This technique allows investigating molecular details by enlarging the samples features thanks to a protocol of hydrogel embedding and subsequent specimen swelling [9].

Photodamage and photobleaching are key aspects that any microscopist faces when working with live samples. The use of red-shifted and near-infrared wavelengths ensures a reduced phototoxicity compared to visible and UV wavelengths, and the intrinsic optical sectioning of multiphoton excitation prevents photobleaching out of the focal plane. However, the need for high photon density in the focal volume must be considered, as photobleaching and photodamage are nonlinear processes [31]. A great advantage of SHG and THG imaging is the absence of photobleaching during the prolonged acquisition protocols if you limit the analysis to an intrinsic molecule. However, these techniques allow the analysis of a limited number of molecular species, and their combination with fluorescent staining must consider the bleaching of fluorophores during the acquisition.

Technical advances in this direction would open new possibilities for label-free microscopy in the field of regenerative medicine and tumor diagnosis. A very promising improvement consists of label-free SHG microendoscopy systems thanks to turn-key Ti:Sapphire lasers coupled to optic fibers possibly enabling the analysis of collagen bundles with possible applications to cancer diagnosis at earlier stages.

## Figures and Tables

**Table 1:** Fields of application of label-free multiphoton microscopy.

Biomedical Field	Representative Results
Regenerative medicine and tissue engineering	- The decellularization process monitored combining SHG of collagen scaffold with autofluorescence or labeling to confirm the absence of cellular component [40].
	- Decellularization maintenance of collagen structure, orientation, and other physical properties [39].
Cancer	- Identification of metastasis toward diagnosis [45].
	- Role of microenvironment and stroma–tumor interface [46].
	- Influence of collagen structure in cancer progression [33,47].
	- Preclinical humanized models of bone marrow niche in leukemia [48,49].
Cardiovascular	- Evaluation of atherosclerotic plaque [50,51,52].
	- Analysis of collagen deposition in infarcted area [53].
Neuroscience	- Myelination in the central nervous system [54,55].
	- Senile plaque in Alzheimer’s model [56].
In vitro 3D models	- Self-assembled fibrillar gels imaged with SHG to study metastatic invasion [41].
	- Scaffold based in vitro model of bone marrow niche [48].
Development and embryogenesis	- Mouse cardiac organogenesis [57].
	- Nematode embryogenesis in C. Elegans [58].
Immunology	- Leukocytes behavior in tumor microenvironment [59].
	- Tumor cell invasion [33].
Ophthalmology	- Imaging of the cornea to diagnose dystrophies and endothelial dysfunctions [60].
Respiratory disease	- Collagen microstructure in interstitial pneumonia [61].
	- Collagen deposition in inflammation driven mouse model of chronic obstructive pulmonary disease [62].
Muscle physiology and pathology	- Fiber-type discrimination with NADH autofluorescence [11,63].
	- Muscle striated features [64].
Kidney, Colon, and Liver	- Tubulointerstitial fibrosis and glomerulosclerosis [65].
	- Endomicroscopy of murine colon mucosa [66].
	- Collagen deposition in liver fibrosis and cirrhosis [67].

**Table 2:** Features and the approaches employed for image analysis and the corresponding degree of difficulty for the two-photon microscope sophistication and data analysis complexity. + = low difficulty; ++ = medium difficulty; and +++ = high difficulty.

Features	Methods	Set-Up Sophistication	Analysis of Complexity
Amount and texture description	First-order statistics (FOS)	+	+
	Second-order statistics (gray level co-occurrence matrix, GLCM)	+	++
	2D-Fast Fourier Transformation (2D-FFT)	+	++
	Wavelet transformation	+	+++
Fibers orientation	Forward–backward SHG signal (F-SHG/B-SHG)	++	++
	Polarization	+++	++
	Coherency (C)	+	+++
Fibers waviness	Straightness parameter (Ps)	+	++
Fibers thickness and distance	Plot profile	+	+

**Table 3:** First-order statistics (FOS) parameters with the corresponding meaning and the interpretation.

Parameters	Meaning	Interpretation in the Image
Mean	Average value	The average value of gray tones
Standard Deviation	The standard deviation of the gray values used to generate the mean gray value	Contrast
Integrated Density	Product of the image’s area and mean gray value	Lightness/darkness
Skewness	It quantifies how symmetrical the distribution is relative to the mean value	The imbalance between the extent of areas (or number of pixels) that are darker or brighter than the mean
Kurtosis	It quantifies whether the shape of the data distribution matches the Gaussian distribution	The spread of gray tones around the mean value

**Table 4:** Second-order statistics (gray level co-occurrence matrix, GLCM) parameters with the corresponding meaning and the interpretation.

Parameters	Meaning	Interpretation in the Image
Inverse difference moment (IDM)	Quantifies the local similarities present in the image	Homogeneity
Energy	Probabilities of different gray levels in the image	Uniformity
Inertia	The similarity in gray levels between neighboring pixels	Contrast
Entropy	Measure the lack of spatial organization inside the image	Randomness
Correlation	Dependence of gray levels between two pixels separated by a certain distance	Regularity in repetition patterns

## Data Availability

Not applicable.

## Abbreviations

STED	Stimulated Emission Depletion
STORM	Stochastic Optical Reconstruction Microscopy
PALM	Photo Activated Localization Microscopy
NADH	1,4-DiHydroNicotinamide Adenine Dinucleotide
SHG	Second Harmonic Generation
THG	Third Harmonic Generation
CARS	Coherent Anti-Stokes Raman Spectroscopy
SRS	Stimulated Raman Spectroscopy
MPM	Multiphoton Microscopy
TPE	Two-Photon Excitation
OPO	Optical Parametric Oscillator
MPF	Multiphoton Fluorescence
LFM	Label-Free Multiphoton
YFP	Yellow Fluorescent Protein
FAD	Flavin Adenine Dinucleotide
FA	Formaldehyde
PFA	Paraformaldehyde
FOS	First-Order Statistics
GLCM	Gray Level Co-Occurrence Matrix
FFT	Fast Fourier Transformation
IDM	Inverse Difference Moment
iSCAT	Interferometric Scattering
PSHG	Polarization-Sensitive Second Harmonic Generation
